# A historical cohort study of eosinophilic inflammation in chronic rhinosinusitis with nasal polyps in Okayama, Japan

**DOI:** 10.1186/2045-7022-3-S2-O3

**Published:** 2013-07-16

**Authors:** Misato Hirai, Mitushiro Okano, Yasuyuki Noyama, Takenori Haruna, Kazunori Nishizaki

**Affiliations:** 1 Okayama Saiseikai General Hospital, Okayama, Japan; 2 Okayama University Graduate School of Medicine, Dentistry and Pharmaceutical Sciences, Department of Otolaryngology-Head & Neck Surgery, Okayama, Japan

## Background

CRSwNP(Chronic rhinosinusitis with nasal polyps) is characterized with eosinophil infiltration into sinonasal tissues in Caucasian patients. In Japan, this condition was thought to be an infectious disease (so called "empyema") for a long time. However, after a clinical profile of ECRS(eosinophilic chronic rhinosinusitis) was first introduced by Moriyama in 2002, the prevalence of ECRS in CRSwNP seems to be increasing in clinical setting. In the present study, we examined a historical cohort study and determined the alteration of eosinophilic inflammation in sinonasal tissues in Japanese CRS.

## Method

Specimens of the sinonasal tissues from adult patients with CRSwNP were collected at the time of paranasal sinus surgery. We selected surgery specimens between 1961 and 1984 (Group A: n=100) and all subjects in 2012 (Group B: n=104 ), for a comparative assessment used historical cohort study. The lamina propia just beneath the epithelial layer was observed under a light microscope, and the number of infiltrated eosinopils per visual field at ~400 magnification was counted.

## Result

The number of eosinophils infiltrating the nasal or paranasal sinus mucosa was significantly larger Group B (2012) than Group A (1961-1984).

## Conclusion

This result was consisted with the report that patients with ECRS have been increased in Japan. The increase of co-morbidity including allergic rhinitis or bronchial asthma may cause an increase of patients with ECRS. Secondly, macrolide therapy for CRS became popular in the previous two decades in Japan. Therefore, the decrease of infectious CRS which requires surgical treatment may affect the increase of ECRS.

